# Coping Strategies and Suicidality: A Cross-Sectional Study From China

**DOI:** 10.3389/fpsyt.2020.00129

**Published:** 2020-03-13

**Authors:** Jianqiang Liang, Kairi Kõlves, Bob Lew, Diego de Leo, Lu Yuan, Mansor Abu Talib, Cun-xian Jia

**Affiliations:** ^1^School of Human Services and Social Work, Griffith University, Logan, QLD, Australia; ^2^World Health Organization Collaborating Centre for Research and Training in Suicide Prevention, Australian Institute for Suicide Research and Prevention, Griffith University, Brisbane, QLD, Australia; ^3^Department of Social Psychology, Faculty of Human Ecology, Putra University of Malaysia, Seri Kembangan, Malaysia; ^4^Department of Epidemiology, School of Public Health, Shandong University, Jinan, China; ^5^Shandong University Centre for Suicide Prevention Research, Shandong University, Jinan, China; ^6^Department of Human Development and Family Study, Putra University of Malaysia, Seri Kembangan, Malaysia

**Keywords:** China, coping skills, suicidal behavior, mental health, students

## Abstract

**Background and Objective:** Suicide is a leading cause of death in young people. Suicidal thoughts and behaviors can be triggered by life and study stresses; therefore, it is important to understand the role of coping strategies. The current study analyzed the link between different coping strategies and suicidality in university students in China.

**Methods:** A cross-sectional study of 2,074 undergraduate students from China used a stratified-clustered-random sampling method (response rate 94.4%). The Suicidal Behaviors Questionnaire–Revised Scale was used to identify suicidal risks, while the Brief COPE scale was used to measure different coping strategies. Univariate and multivariate logistic regression analyses were utilized to examine coping strategies and suicidality.

**Results:** A negative association of some coping skills (active coping and positive reframing) with suicidality and a positive association of some other coping skills (self-distraction, substance abuse, behavioral disengagement, venting, and self-blame) with suicidality were observed after adjusting for sociodemographic and mental health variables.

**Conclusions:** Training and supporting young people to identify and apply adaptive coping strategies to deal with life stress could help to reduce suicidal ideation and behavior.

## Introduction

Suicidal behavior in young people is a public health and social issue globally (1–3). Suicide is a leading cause of death in young people (4); furthermore, nonfatal suicidal behavior is more prevalent in younger age groups (5, 6). Similarly to other countries, China is reporting a high prevalence of suicidal behavior among young people, including college students (7–9). Transitioning from adolescence to young adults, university students are considered as the future elite; however, academic and career expectations from themselves and their parents, competitive environments, achieving less than hoped, and failing a grade may lead to interpersonal conflicts and a sense of isolation, and further to stress, low personal control, autistic traits, internet addiction, depression, anxiety, and suicidal thoughts and attempts (10–15).

There is currently limited knowledge about protective factors for suicidal behavior and ideation. Existing research has examined various coping strategies impacting suicidal behaviors (16) and has analyzed gender-specific coping strategies associated with suicidal ideation in university students (17). Nevertheless, there has been relatively limited research that has focused on protective and risk effects of specific coping styles (18–20).

The available literature is inconsistent in conceptualizing and categorizing different coping styles (21, 22). This might be partly attributable to a number of different scales used to investigate coping strategies in various social and cultural contexts, such as the COPE (23), the Brief COPE (24), and the Coping Styles Questionnaire (CSQ) (25). Nevertheless, one common view is that individuals would apply adaptive and/or maladaptive (dysfunctional) coping skills when they face stress and threats (23, 26, 27). A recent study using Brief COPE from China showed that having a meaning of life is a protective factor, while self-distraction and self-blame are risk factors for suicidality in collage students (28). In another cross-sectional study, Zhang et al. (9) used CSQ to evaluate suicidality of university students in China. They found that passive coping (especially fantasizing) was positively associated with suicidal ideation. The results of Horwitz et al. (29), based on a cross-sectional study using Brief COPE, showed that behavioral disengagement and self-blame increased suicidal vulnerability. Furthermore, in a prospective study in college students using COPE, Chou et al. (30) reported that ineffective coping skills together with persistence stress and negative emotions could generate higher risk of suicidality.

Research has also shown mixed results regarding the relationship between avoidant coping and suicidality. Some studies indicate that avoidance coping strategies heighten the risk of suicidality as individuals tend to resign themselves to the problem, and do not undertake further efforts to reduce stressors (31–33). Others suggest that avoidance coping with a good purpose (such as decreasing the negative feelings of life stressors and temporary shifting focus from the stressors to other important things in life) could prevent suicidal behavior in adolescents and young people (20, 34). Nevertheless, none of the studies above used Brief COPE in measuring avoidant coping. It is important to better understand the relationship between different coping strategies (particularly avoidance strategies) and suicidality in young people. Therefore, this paper aims to analyze the association between specific coping strategies and suicidality (suicidal ideation and behavior) in a sample of university students in China.

## Methods

### Participants and Recruitment

In November 2016, undergraduate university students from two universities in Jinan (the capital city of Shandong province), China, voluntary participated in a cross-sectional survey. In total 2,074 students responded, with a response rate of 94.4%. A stratified–clustered–random sampling method was applied to select from three or four classes of students for each grade. The study received ethics approval from the institutional review board of the Ethics Committee at the School of Public Health, Shandong University (No. 20161103). Each participant signed an informed consent form before undertaking the questionnaire. The survey contained a brief battery of self-report psychosocial instruments, as described below.

### Scales

The Suicidal Behaviors Questionnaire Revised Scale (SBQR) (35), adapted from Osman et al. (36), contains four items that assess suicidal thoughts and behaviors from different perspectives: lifetime suicidal ideation and attempt(s), frequency of suicidal ideation over the past year, risk of suicide attempt, and likelihood of suicidal behavior in the future. The Chinese version of the SBQ-R was validated (35) and adapted for this study. The total score of the scale was calculated, and a cut-off value of 7 was applied as recommended by Osman et al. (36).

Depression, Anxiety & Stress Scale (DASS-21) (37) is a 21-item scale that measures three dimensions of mental health: depression (Cronbach's alpha = 0.84), anxiety (Cronbach's alpha = 0.79), and stress (Cronbach's alpha = 0.82). Responses for all items range from “0—Did not apply to me at all” to “3—Applied to me very much or most of the time.”

The Brief COPE Scale consists of 28 items to measure 14 different coping strategies (24). In this study, the Chinese version of the scale (38) was adapted. The scale presents good internal reliability, with Cronbach's alpha = 0.83. The 14 dimensions of coping strategies are comprised of self-distraction, active coping, denial, substance abuse, use of emotional support, use of instrumental support, behavioral disengagement, venting, positive reframing, planning, humor, acceptance, religion, and self-blame. Each dimension contains two items, with sum score as the total dimension score.

The questionnaire also included sociodemographic categories, such as gender, ethnicity, residency, being an only child (with no siblings), general physical health and mental health, academic performance, family economic status, parent education level, parental bonding, and religious affiliation.

### Statistical Analysis

Odds ratios (ORs) with 95% confidence intervals (CI) were calculated to explore differences between the suicidal and non-suicidal groups by the categorical sociodemographic variables. Independent sample *t*-tests were used to identify differences between the two groups by coping strategies, and effect size (Cohen's d) was calculated for each coping skill. The Cohen's d cut-off values were considered as small (≥0.2), medium (≥0.5), and large (≥0.8) (39). Further, each coping skill was entered as a predictor into a logistic regression with suicidality (suicidal vs. non-suicidal) as the dependent variable. The calculations were adjusted for confounding effect of other factors: (1) a coping skill + sociodemographic variables; (2) a coping skill + sociodemographic variables + Depression + Anxiety + Stress. A probability level of 0.05 was applied for all statistical tests. SPSS version 22.0 was used for data analysis (IBM SPSS, Inc. in Chicago, Illinois, USA).

## Results

Among the 2,074 students, 20.6% (*n* = 428) were identified to have an SBQ-R total score of 7 or above and were included in the suicidal group, and the remaining 79.4% (*n* = 1,646) were assigned to the non-suicidal group. Table 1 presents the sociodemographic backgrounds of participants in the suicidal and non-suicidal groups. Gender, being an only child, and religious affiliation did not differ significantly between the suicidal and non-suicidal groups. However, there were significant differences on residency, academic performance, and family economic status between the suicidal and non-suicidal groups. Students from urban areas, with poor academic performance, and from families with poorer socioeconomic background were more likely to be suicidal (see Table 1).

**Table 1:** Sociodemographic background of non-suicidal group and suicidal group in the study.

	**Suicidal (*****n*** **=** **428)**		**Non-suicidal (*****n*** **=** **1,646)**		**OR**	**95% CI**	
	***N***	**%**	**N**	**%**		**Lower**	**Upper**
**Gender**							
Female	290	67.8	1,078	65.5	1.22	0.96	1.55
Male	138	32.2	568	34.5	1		
**Residency**							
Urban	225	52.6	790	48.0	**1.39**	**1.08**	**1.80**
Rural	203	47.4	856	52.0	1		
**Only child**							
Yes	213	49.8	799	48.5	1.01	0.78	1.31
No	215	50.2	847	51.5	1		
**Academic performance**							
Poor	90	21.0	172	10.4	**2.17**	**1.62**	**2.91**
Neutral	291	68.0	1185	72.0	1		
Good	47	11.0	289	17.6	0.74	0.52	1.05
**Family economic status**							
Very poor	9	2.1	47	2.9	0.68	0.32	1.44
Poor	76	17.8	213	12.9	**1.40**	**1.03**	**1.90**
Neutral	301	70.3	1118	67.9	1		
Good	38	8.9	239	14.5	**0.61**	**0.41**	**0.89**
Very good	4	0.9	29	1.8	0.62	0.21	1.83
**Religious affiliation**							
Yes	26	6.1	106	6.4	0.84	0.53	1.32
No	402	93.9	1540	93.6	1		

*Results in bold indicate to a significance level of 0.05*.

As shown in Table 2, there were significant differences between the suicidal and non-suicidal groups for all coping strategies except “Use of emotional support.” The effect size of the coping strategies were between a small and medium level. Table 3 presents ORs and 95% CIs after adjusting for the sociodemographic variables (gender, residency, being an only child, academic performance, family economic status, and religious affiliation). Self-distraction [Adj' OR = 1.09, 95%CI (1.01–1.18)], denial [Adj' OR = 1.23, 95% CI (1.14–1.33)], substance abuse [Adj' OR = 1.33, 95% CI (1.23–1.43)], behavioral disengagement [Adj' OR = 1.32, 95% CI (1.22–1.43)], venting [Adj' OR = 1.10, 95% CI (1.02–1.18)], humor [Adj' OR = 1.17, 95% CI (1.09–1.26)], religion [Adj' OR = 1.15, 95% CI (1.07–1.24)], and self-blame [Adj' OR = 1.28, 95% CI (1.18–1.38)] were significantly more likely to be used by students in the suicidal group. Active coping [Adj' OR = 0.77, 95% CI (0.71–0.83)], use of instrumental support [Adj' OR = 0.90, 95% CI (0.84–0.97)], positive reframing [Adj' OR = 0.82, 95% CI (0.76–0.89)], planning [Adj' OR = 0.84, 95% CI (0.77–0.91)], and acceptance [Adj' OR = 0.90, 95% CI (0.83–0.97)] were significantly less likely to be used by the suicidal group.

**Table 2:** Suicidal and non-suicidal group by copying styles (as per the Brief COPE Scale).

**Coping style**	**Suicidal (*n* = 428)**	**Non-suicidal (*n* = 1,646)**			**Effect size**
	**M (SD)**	**M (SD)**	**t (df)**	***p*-value**	**(Cohen's d)**
Self-distraction	5.92 (1.42)	5.74 (1.44)	−2.22 (2072)	**0.026**	0.126
Active coping	6.13 (1.40)	6.67 (1.33)	7.44 (2072)	**<0.001**	0.395
Denial	4.01 (1.49)	3.59 (1.33)	−5.71 (2072)	**<0.001**	0.297
Substance use	3.22 (1.68)	2.68 (1.27)	−6.23 (560.7)^a^	**<0.001**	0.363
Use of emotional support	5.61 (1.58)	5.57 (1.51)	−0.44 (2072)	0.659	0.026
Use of instrumental support	5.76 (1.54)	5.97 (1.46)	2.54 (638.7)^a^	**0.011**	0.140
Behavioral disengagement	4.11 (1.43)	3.52 (1.35)	−7.95 (2072)	**<0.001**	0.424
Venting	5.47 (1.42)	5.26 (1.51)	−2.50 (2072)	**0.012**	0.143
Positive reframing	5.76 (1.47)	6.21 (1.44)	5.69 (2072)	**<0.001**	0.309
Planning	6.04 (1.30)	6.40 (1.33)	5.05 (678.7)^a^	**<0.001**	0.274
Humor	4.44 (1.57)	4.07 (1.48)	−4.40 (637.7)^a^	**<0.001**	0.243
Acceptance	6.06 (1.33)	6.27 (1.32)	3.00 (2072)	**0.003**	0.158
Religion	3.78 (1.48)	3.52 (1.41)	−3.43 (2072)	**0.001**	0.180
Self-blame	5.45 (1.39)	4.94 (1.42)	−6.64 (2072)	**<0.001**	0.363

a *Equal variances not assumed*.

*Results in bold indicate to a significance level of 0.05*.

**Table 3:** Multivariate logistic regression analyses of the association between copying skills and suicidality (suicidal vs. non-suicidal) adjusted for sociodemographic factors and mental health.

**Coping style**	**Adj' OR^**a**^**	**95%CI**		**Adj' OR^**b**^**	**95%CI**	
		**Lower**	**Upper**		**Lower**	**Upper**
Self-distraction	**1.09**	**1.01**	**1.18**	**1.11**	**1.02**	**1.20**
Active coping	**0.77**	**0.71**	**0.83**	**0.86**	**0.79**	**0.94**
Denial	**1.23**	**1.14**	**1.33**	1.08	1.00	1.18
Substance use	**1.33**	**1.23**	**1.43**	**1.17**	**1.08**	**1.27**
Use of emotional support	1.02	0.95	1.09	0.99	0.92	1.07
Use of instrumental support	**0.90**	**0.84**	**0.97**	0.94	0.87	1.02
Behavioral disengagement	**1.32**	**1.22**	**1.43**	**1.13**	**1.04**	**1.24**
Venting	**1.10**	**1.02**	**1.18**	**1.08**	**1.00**	**1.17**
Positive reframing	**0.82**	**0.76**	**0.89**	**0.90**	**0.83**	**0.98**
Planning	**0.84**	**0.77**	**0.91**	0.92	0.84	1.00
Humor	**1.17**	**1.09**	**1.26**	1.06	0.98	1.15
Acceptance	**0.90**	**0.83**	**0.97**	0.97	0.89	1.06
Religion	**1.15**	**1.07**	**1.24**	1.02	0.94	1.11
Self-blame	**1.28**	**1.18**	**1.38**	**1.16**	**1.07**	**1.26**

a *Adjusted for sociodemographic variables*.

b *Adjusted for sociodemographic variables, and depression, anxiety, stress (DASS-21)*.

*Results in bold indicate to a significance level of 0.05*.

Further adjustment for the sociodemographic variables and three dimensions of the DASS-21 (depression, stress, anxiety) showed that self-distraction [Adj' OR = 1.11, 95% CI (1.02–1.20)], substance abuse [Adj' OR = 1.17, 95% CI (1.08–1.27)], behavioral disengagement [Adj' OR = 1.13, 95% CI (1.04–1.24)], venting [Adj' OR = 1.08, 95% CI (1.00–1.17)], and self-blame [Adj' OR = 1.16, 95% CI (1.07–1.26)] remained significantly associated in a positive direction with suicidality. Active coping [Adj' OR = 0.86, 95% CI (0.79–0.94)] and positive reframing [Adj' OR = 0.90, 95% CI (0.83–0.98)] remained negatively associated with suicidality (Table 3).

## Discussion

The present study aimed to better understand the association between specific coping strategies and suicidality using the Brief COPE scale and measures of suicidality in a sample of Chinese university students. A significant association was found. More specifically, after controlling for sociodemographic circumstances, stress, depression, and anxiety, the strategies of active coping and positive reframing were less likely to be used by the suicidal group compared to the non-suicidal group. Self-distraction, substance abuse, behavioral disengagement, venting, and self-blame were more likely to be used by the suicidal group.

Active coping has been found to buffer suicidality (9, 40), and a similar effect has also been shown for positive reframing (19, 41). These are both adaptive coping skills, with active coping referring to actively removing or reducing the stressors, and positive reframing to cognitively constructing a stressful transaction in a positive way (23). The findings of this study suggest that the application of these two coping skills could be beneficial to students in reducing their suicidality. Furthermore, the use of instrumental support, planning, and acceptance were also negatively associated with suicidality after controlling for sociodemographic circumstances, indicating that these coping strategies may also be helpful in reducing suicidality in young people (42).

Several coping strategies related to avoidance, such as behavioral disengagement, self-distraction, venting, and humor, were more likely to be used by suicidal students in the current study; however, previous research has not clearly specified the adaptive or maladaptive effect of these strategies in dealing with stress (23, 43) or suicidality (20). Behavioral disengagement was positively associated with suicidality in our study, echoing results from some earlier studies in late adolescence (29) and in university students (44). Nevertheless, some scholars argue that behavioral disengagement could help reduce the risk of suicidality by temporarily shifting focus away from the stressors, to release negative emotions, and finally to turn back to problem-solving (20, 34).

The results showed that self-blame and use of alcohol and drugs to cope with problems and escape from stress (“substance use” by the Brief COPE scale) were significantly more likely to be used by suicidal students and also showed the highest adjusted OR after controlling for the confounding effects of sociodemographic circumstances, stress, depression, and anxiety. Several studies have demonstrated an association between alcohol and substance use in suicidality (45, 46). Drink to cope could associate with poor problem-solving skills, avoidance coping, and negative urgency in young people who have suicidal risk (47). In addition, a rapid increase in alcohol use in China and alcohol consumption becoming more normalized in recent decades call for further public health actions (48).

Although having a religious affiliation was not significantly different in suicidal and non-suicidal groups, contrary to our expectations, we found “religion” as a coping strategy to be significantly more frequent in the suicidal group. Nevertheless, after controlling for sociodemographic factors, stress, anxiety, and depression, this association became non-significant. It is also important to note that in the Brief COPE scale, the items under “religion” also include spirituality. Cook (49) indicates that there have been mixed results on this topic and argues that those who are spiritual but not religious may not experience buffering or protection against depression or suicidal behavior. Furthermore, remarkable differences in the relationship between religion and suicidality between countries have been reported (50).

It is important to note some of the limitations of this study. Despite a large sample size, a relatively small number of students were identified as having experienced suicidal ideation and attempted suicide. Therefore, we were unable to separately analyze the differences between those who experienced suicidal ideation and those who (also) attempted suicide. The Brief COPE scale helped articulate the coping strategies that the students preferred/applied; however, it is not necessarily comparable with other studies that used different coping scales. Furthermore, the cross-sectional nature of the study limits the analyses to the association between coping styles and suicidality, and does not allow us to determine causality, and we cannot present the effectiveness of each coping strategy (51). It is also important to note that we merged suicidality as measured by SBQ-R, and therefore it may not be applicable specifically to the risk of suicide attempts or suicides. Lastly, the research participants were university students, and therefore the results are not generalizable to all young people in China.

Nevertheless, these results may present some important implications. Using active coping skills to solve problems and developing a positive self-appraisal (52) have been shown to be important for young people facing stressful events and may help to reduce their suicide risks. It is also important to encourage help-seeking from families, peers, and other professionals while having suicidal thoughts (6). In addition, we could help young people to identify the negative impacts of maladaptive coping strategies (such as using drugs or alcohol to manage stress) and replace them with more adaptive coping strategies to reduce the risks of suicide. Programs and services, such as cognitive behavioral therapy (2), mindfulness training to manage stress (53), and routine counseling screening to identify the individuals who rely heavily on avoidance coping (33), could be put in place for young people suicide prevention. The student-friendly university mental health services could also be helpful in connecting students with broader public health resources, and in educating them to cope effectively with academic and life stress (54).

## Conclusion

This study provides new insights on the relationship between coping strategies and suicidality in young people. After controlling for the possible confounding effects of sociodemographic circumstances, stress, depression, and anxiety, active coping and positive reframing were negatively associated with suicidality, whereas self-distraction, substance abuse, behavioral disengagement, venting, and self-blame were positively associated with suicidality. There is a need to support young people to develop adaptive and effective coping strategies in order to reduce suicide ideation and attempts.

## Data Availability Statement

The datasets used and/or analyzed during the current study are available from the corresponding author on reasonable request.

## Ethics Statement

The studies involving human participants were reviewed and approved by the institutional review board of the Ethics Committee at the School of Public Health, Shandong University (No. 20161103). The patients/participants provided their written informed consent to participate in this study.

## Author Contributions

BL, CJ, and KK designed the study. CJ, LY, and BL organized data collection. JL and KK analyzed and interpreted the data. JL and KK were major contributors in writing the manuscript. All authors read and approved the final manuscript.

### Conflict of Interest

The authors declare that the research was conducted in the absence of any commercial or financial relationships that could be construed as a potential conflict of interest.

## Abbreviations

WHO: World Health Organization
SBQ-R: The Suicidal Behaviors Questionnaire–Revised Scale
DASS-21: Depression, Anxiety & Stress Scale
OR: odds ratio
CI: confidence intervals.
